# Breast magnetic resonance imaging (MRI) surveillance in breast cancer survivors

**DOI:** 10.1186/s40064-015-1158-5

**Published:** 2015-08-28

**Authors:** Chana Weinstock, Cristina Campassi, Olga Goloubeva, Kathleen Wooten, Susan Kesmodel, Emily Bellevance, Steven Feigenberg, Olga Ioffe, Katherine H R Tkaczuk

**Affiliations:** University of Maryland Greenebaum Cancer Center, 22 South Greene Street, RM S9D, Baltimore, MD 21201 USA

**Keywords:** Breast cancer, Magnetic resonance imaging, Bilateral, Surveillance

## Abstract

**Purpose:**

As the breast cancer survivor population increases, the topic of screening these women for recurrences is increasingly relevant. In our institution, we use both breast MRI and mammography in the surveillance of breast cancer survivors, although little data exists on the use of MRI in this setting. We present a retrospective analysis of our experience and compare the sensitivity and specificity of MRI vs. mammography in this setting.

**Methods:**

We identified women under 65 with a history of breast cancer and at least one follow-up MRI performed along with a mammogram done within 6 months of the MRI. We compared the outcomes of MRI and mammography in terms of biopsies performed as well as in detection of new cancers.

**Results:**

Of 617 charts reviewed, 249 patients met inclusion criteria, with 571 paired MRI/mammogram results. There were 27 biopsies performed due to MRI findings alone, 10 done due to mammographic findings alone, and 15 done based on abnormalities seen on both imaging modalities. There were 8 malignancies identified based on an abnormal MRI, 3 detected on both MRI and mammography, and none identified via mammography alone. Overall, MRI had a sensitivity of 84.6% (the 95% CI 54.6–98.1) and a specificity of 95.3% (the 95% CI 93.3–96.9); mammography a sensitivity of 23.1% (the 95% CI 5.0–53.8), and a specificity of 96.4% (the 95% CI 94.5–97.8).

**Conclusions:**

Breast MRI is a useful surveillance modality in breast cancer survivors and may be more sensitive at detecting recurrences than mammography alone in this population.

## Background

Breast cancer remains one of the most common cancers affecting women in the United States, with an annual incidence of approximately 232,670 cases forecasted for 2014. As early diagnosis rates and treatments improve, the breast cancer survivor population continues to increase, with an estimated 2.9 million women in the US currently alive with a personal history of breast cancer (2014). The question of how to best follow these patients once they have completed therapy for their initial breast cancers is becoming increasingly relevant.

Women with a history of breast cancer are known to face a higher risk of developing new or recurrent disease. Estimated risk of contralateral tumor development in breast cancer survivors who have completed initial therapy and have not undergone prophylactic contralateral mastectomy is approximately 0.5% per year and is not dependent on age of diagnosis or time since diagnosis (Chen et al. 1999), although there is some evidence that this risk is decreasing in the era of modern systemic therapy (Nichols et al. 2011).

At this point there remains much controversy regarding the optimal imaging modality to use in surveillance of these women for the development of contralateral breast cancers or of in-breast recurrences. Current guidelines published by the American Society of Clinical Oncology recommend mammography alone as an imaging modality suitable for surveillance of these patients; this is similar to the recommendation for the use of mammogram in the routine surveillance of unaffected women over 50. No other imaging modality, including breast magnetic resonance imaging (MRI), was thought to have sufficient evidence to justify inclusion in the recommended surveillance of breast cancer survivors (Khatcheressian et al. 2012). NCCN guidelines similarly recommend annual mammography as the mainstay of surveillance for these patients (2014).

When stating that MRI is not recommended in surveillance of breast cancer survivors it is useful to examine the evidence base for the use of the recommended surveillance mode, i.e. mammography. In actuality, there is no randomized clinical trial that examines the impact on breast cancer mortality achieved via the use of annual mammography, as recommended, in breast cancer survivors. While there is some suggestion of benefit based on systematic review of small data sets (Robertson et al. 2011), the use of mammography in the breast cancer survivor cohort is partially based on extrapolation of likely benefit from trials of population screening which have demonstrated a reduction breast cancer mortality (Houssami and Ciatto 2010).

Extrapolating data on benefit from population studies is problematic since breast cancer survivors may differ in several important ways from the population at large. Namely, breast cancer survivors tend to be younger, with approximately 20% of all new cases in the U.S. diagnosed in women under 50 (2014). In most primary screening studies, the reduction in mortality conferred by screening mammography was less for women ages 40–49 than for women over age 50 (Humphrey et al. 2002). Young women tend to have dense breasts, thus decreasing mammographic sensitivity (Wolfe 1976; Carney et al. 2003). Additionally, risk factors that played a role in the development of first cancers, whether they are environmental or genetic or a combination of the two, are still relevant in raising the risk that breast cancer survivors face in developing new or recurrent cancers. There are also risks of local recurrence of treated breast tumors that the general population does not face.

For all of the above reasons, breast cancer survivors tend to be at a higher baseline risk of tumor occurrence, and screening recommendations that apply to the general population may not be adequate. In fact, systematic review of the literature in the retrospective setting indicates that when recurrent or new breast cancers are detected in patients who have a history of breast cancer, they may be detected via methods other than mammography. Previous studies have reported that 50–72% of ipsilateral recurrences (Orel et al. 1992; Grosse et al. 1997; Ashkanani et al. 2001; Joseph et al. 1998) and 37–80% of contralateral new primaries (de la Rochefordière et al. 1996; Kollias et al. 2000; Hill-Kayser et al. 2006; Robinson et al. 2007; Weinstock et al. 2012) are mammographically-detected.

The empiric use of MRI as an adjunct to mammography for surveillance of breast cancer survivors (Wernli et al. 2014) is also an extrapolation, based on data obtained via the use of this imaging modality in other high-risk patient populations, such as in the screening of BRCA1 and BRCA2 mutation carriers (Kuhl et al. 2005; Warner et al. 2004; Kriege et al. 2004; Leach et al. 2005; Lehman et al. 2005). In this setting, MRI has proven to be useful in detecting what appear to mammographically-occult cancers, and is in fact recommended for routine screening of these patients as a result (2014). There is data demonstrating clinical utility and high cancer detection rate in the use of MRI in this setting in a formalized, population-based screening program (Chiarelli et al. 2014). Conversely, there has been recent evidence that the use of MRI in another setting; i.e. in the preoperative imaging of the affected and contralateral breast in newly-diagnosed patients, may be less useful than originally thought in terms of its effect on local or distal recurrence risks (Houssami et al. 2014).

In view of the uncertainty of the utility of breast MRI in those with a personal history of breast cancer, and in light of the paucity of published data on what is becoming an ever-enlarging cohort of patients, we felt that a review of our institutional data would be useful to add to the literature on the subject. At our institution we have been using MRI since 2005 as an adjunct to mammography in surveillance of these patients. We therefore present a review of our experience at the University of Maryland Medical Center in the use of MRI in screening women with a personal history of breast cancer. Our study aimed to compare the detection rate of ipsilateral recurrences and/or of contralateral new primaries in breast cancer survivors under 65 screened at our institution using MRI as an adjunct to mammography. We also aimed to compare the false-positive rate of surveillance MRI in this cohort to that of mammography, as well as to determine the rate of additional biopsies necessitated by the addition of MRI to routine mammography.

## Methods

After approval was obtained by the institutional review board, we identified all consecutive breast MRIs performed at the University of Maryland Medical Center from January 1, 2005 through December 31, 2011. We used chart reviews of electronic medical records for each patient with a history of breast cancer to retrospectively identify asymptomatic breast cancer survivors imaged with breast MRI within 6 months of a mammogram,. We excluded MRIs done at presentation or within 11 months of initial cancer diagnosis. We excluded patients with stage IV disease and patients with imaging performed for diagnostic purposes for abnormal clinical findings. Additionally excluded were patients who had undergone bilateral mastectomy and those who were known BRCA1 or BRCA2 mutation carriers. Lastly, we excluded all MRIs done on those with a history of high-risk breast lesions only, such as lobular carcinoma in situ (LCIS), and studies done on patients above age 65. Male patients were also excluded.

Once appropriate MRI/mammography pairs were identified, we conducted a chart review of the electronic medical records of identified patients to further obtain clinical data. Patient’s on-study characteristics included patient age, race, stage at original diagnosis, as well as type of original surgery performed (mastectomy vs. lumpectomy), TNM stage of original tumor, and ER/PR and Her-2 status of original tumors. We then looked at data on each MRI and mammography performed in follow-up for each patient and recorded data on the date of each study and the Breast Imaging Reporting and Data System (BI-RADS) (D’Orsi et al. 2003) score assigned each study by the reading radiologist.

All mammograms were performed on Hologic equipment, which was digital after 2007, and included initial standard CC and MLO views of each breast or the unilateral remaining breast if the patient had unilateral mastectomy without reconstruction. All mammograms were performed on equipment accredited by the America College of Radiology and meets the requirements for technical parameters and image quality mandated by the Mammography Quality Standard Act of the Food and Drug Administration. All mammograms were interpreted by a fellowship trained breast imager with 2–10 years of experience. Additional supplemental views and/or ultrasounds were performed for as deemed appropriate by the interpreting radiologists.

Breast MRI was performed on a 1.5 or 3 T commercially available system (Siemens) using a dedicated surface breast coil. The breast MRI protocol included a localizing sequence followed by an axial T1 nonfat suppressed sequence (TR = 7 ms, TE = 2.5 ms) and an axial STIR sequence (TR = 4000 ms, TE = 43 ms). Contrast enhanced dynamic imaging included a series of axial gradient echo T1 with fat suppression obtained prior to and following intravenous administration of gadolinium contrast. The three post-contrast dynamic series were obtained at 1, 2, and 6 min. Between the second and third time points, an additional high resolution axial T1 with fat suppression was acquired. The dynamic sequence used TR, 3.9 ms; TE, 1.8 ms; and a flip angle, 10°, slice thickness of 1.2 mm with no gap, a matrix of 448 × 358; and a field of view of 32–40 cm depending on the breast size. Sequence acquisition time was 1 min and 2 s. The high resolution interview sequence used TR, 4.1 ms; TE, 1.9 ms; and flip angle, 10°, slice thickness of 0.8 mm with no gap, a matrix of 448 × 448, and a field of view of 32–40 cm depending on the breast size, the sequence acquisition time was 3 min and 1 s. Unilateral sagittal T2 W series of the right and left breast were also obtained. Total imaging time was approximately 30 min.

Post-processing included Maximum Intensity Projection (MIP) with and without subtraction, and subtracted images obtained by subtracting the dynamic series of theunenhanced images from the first post-contrast. Interpretation was supplemented with Dynacad and Aegis Sentinel software prior to and after 2010, respectively, for further characterization of lesion kinetics.

We reviewed imaging reports for the negative MRIs and mammograms, and BI-RADS score was recorded for each study. Cases with suspicious imaging findings which led to biopsy were retrospectively reviewed in blinded fashion by a fellowship trained breast imager with greater than 10 years of experience. The side, location, number and features of the suspicious imaging findings were recorded according to BI-RADS lexicon. In addition to the breast findings, axillary and extramammary findings were recorded.

Biopsies of BI-RADS 4 and 5 MRIs were performed by a board certified fellowship trained breast radiologist via imaging guided biopsy or by a board certified breast surgeon. The biopsy modality and whether ipsilateral or contralateral to the side of initial breast cancer was recorded for every biopsied lesion. Pathology was reviewed by a board certified pathologist and results were recorded. For malignancies, histology, grade, and staging were recorded. After determining the total number of benign, high risk, and malignant biopsy results, these results were correlated with whether the suspicious findings were detected on MRI, mammogram, or both. In case of ambiguity, review of pathological specimens was retrospectively undertaken by a breast pathologist. Interval cancers detected by physical exam in patients with negative imaging were also recorded.

Once the above data was collected for each patient, sensitivity and specificity were calculated for each modality; mammogram and MRI individually, as well as mammogram combined with MRI. For patients with more than one biopsy, which resulted from a single positive MRI/mammogram pair, all of the biopsies were included in the analysis. For patients with more than one positive MRI/mammogram pair, the second set of exams in the subsequent years and any resulting biopsies were excluded from the analysis so as not to over-represent any single patient in the sample. We then compared the outcome of MRI and mammography in terms of biopsies performed as well as in detection of new cancers.

The following definitions were used for study parameters: True positive (TP) scans were those in which a breast lesion was histologically proven to be an invasive breast cancer or a ductal carcinoma in situ (DCIS). True negative (TN) scans were those without a suspicious lesion seen on imaging and no breast cancer was diagnosed in the next 12 months. False negative (FN) scans were those that did not detect a suspicious breast lesion, but a breast cancer was detected in the following 12 months. False positive (FP) scans were those that resulted in biopsy that was subsequently benign. Sensitivity (Se) was defined as TPR = TP/(TP + FN), and specificity (Sp) was defined as TNR = TN/(TN + FP).

## Results

Of 617 consecutive charts reviewed for patients who underwent MRI at the University of Maryland between 2005 and 2011, 249 patients met inclusion criteria, 124 (49.6%) of whom were African-American. Patient characteristics are presented in Table 1. There were 368 patients found ineligible for study inclusion. Reasons for exclusion are presented in Table 2. There were a total of 571 paired MRI/mammogram results included in our analysis; a median of 2.29 per patient (range 1–6). There were 52 biopsies performed in 43 of the 249 patients in the study (17%) as a result of abnormalities detected on imaging.

**Table 1:** Patients’ on-study characteristics (N = 249)

Ethnicity	N	%
Caucasian	110	44.2
African American	123	49.4
American Indian	3	1.2
Hispanic	3	1.2
Asian	5	2.0
Other	5	2.0
Age at diagnosis, years		
Median	46	
Range	25–64	
Gender		
Female	249	100.0
Surgery		
Lumpectomy	137	55.0
Mastectomy	109	43.4
Unknown	4	1.6
Chemotherapy		
Yes	135	54.2
No	97	39.0
Unknown	17	6.8
Radiation		
Yes	157	63.1
No	78	31.3
Unknown	14	5.6
Hormone therapy		
Yes	152	61.0
No	74	29.7
Unknown	23	9.2
Stage		
0	50	20.1
1	84	33.7
2	69	27.7
3	39	15.7
Unknown	7	2.8
ER		
ER−	45	18.1
ER+	144	57.8
Unknown	60	24.1
PR		
PR−	60	24.1
PR+	119	47.8
Unknown	70	28.1
HER2		
HER2-	103	41.4
HER2+	27	10.8
Unknown	119	47.8

**Table 2:** Reason for study exclusion

Reason for study exclusion	Number (total = 386)
P_t_ > 65	91
MRI done at diagnosis only	60
No mammographic correlate	56
Hx of high-risk abnormality such as LCIS/atypical hyperplasia only	28
Known metastatic or recurrent disease	11
BRCA1 or BRCA2 mutation carrier, Li Fraumeni	5
MRI done <11 months after initial diagnosis	4
Male patient	4
Other, including no personal breast cancer history	127

Overall, 11 patients were diagnosed with malignancy. All 11 malignancies were seen on MRI and 3 were also seen on mammogram. No cancers were detected via mammography alone. Biopsies were done based on abnormalities detected on both MRI and mammography in 15 cases; 20% were malignant (n = 3). There were 27 biopsies done based on abnormalities detected on MRI alone; 30% of the results (n = 8) were malignant. These 8 cancers were detected based on MRI findings and were mammographically occult. There were 10 biopsies performed based on abnormalities detected via mammography alone; the results of all of these biopsies were benign.

The results of the abnormal imaging findings included 47 breast lesions, 4 abnormal axillary lymph nodes, and 1 lung mass. Pathology was obtained in these cases using imaging-guided core needle biopsy (n = 30) and/or surgical biopsy (n = 22). Twelve malignant lesions were detected in 11 of 249 patients overall, at a rate of 4.4%. The 11 cancers detected are described in Table 3, and included 10 breast cancers, 1 metastatic axillary lymph node and 1 isolated lung metastasis. One patient had two in-breast lesions simultaneously detected on the same MRI. Overall, 92% of recurrences were early-stage (Stage 0 or Stage I) disease. Of the 10 in-breast lesions, 50% (n = 5) were ipsilateral recurrences in patients who had undergone breast-conserving therapy (n = 3) or mastectomy (n = 2) and 50% (n = 5) represented contralateral new primaries in patients with prior breast conserving therapy (n = 2) or mastectomy (n = 3).

**Table 3:** Patient characteristics of screen detected cancers

Pt	Original stage	Original surgery	Seen on	Ipsi/Contra	Stage	Age at dx	Age at recurrence
11	I	Lumpectomy	MRI	Contralateral	I	29	48
22	DCIS	Lumpectomy	MRI + mammo	Ipsilateral	II	53	65
33	I	Mastectomy	MRI	Ipsilateral	I	37	44
44	III	Mastectomy	MRI + mammo	Contralateral	I	48	52
55	DCIS	Lumpectomy	MRI + mammo	Contralateral	DCIS	57	61
66	DCIS	Lumpectomy	MRI	Ipsilateral	I	44	52
77	II	Mastectomy	MRI	Contralateral	I	47	55
88	DCIS	Mastectomy	MRI	Ipsilateral	I	44	46
99	III	Lumpectomy	MRI	Ipsilateral	DCIS	44	50
110	III	Mastectomy	MRI	Lung metastases	IV	53	63
111	III	Mastectomy	MRI	Contralateral	DCIS	46	56

Only three of the detected cancers occurred in patients under fifty at the time of follow-up imaging. The median age of patients with recurrences detected in our study was 58.8 (range 44–65). The tumors recurred at a median of 8.6 years following diagnosis of the original cancers (range 2–20). Two of the cancers detected were in the breast remnants of patients who had undergone mastectomy previously. There were two known patients (0.8%) in the study with documented normal MRI and mammograms at our center who were known to have developed interval breast cancers within 6 months of screening, both with stage I disease.

The overall sensitivity and specificity data for MRI and mammography were counted for each round of screening, with an aggregate total calculated for all screening rounds (Table 4). This total includes data from all 571 screens included in the data set. Overall, MRI had a sensitivity of 84.6% (the 95% CI 54.6–98.1) and a specificity of 95.3 (the 95% CI 93.3–96.9). In comparison, mammography had a sensitivity of 23.1% (the 95% CI 5.0–53.8), and a specificity of 96.4% (the 95% CI 94.5–97.8).

**Table 4:** Estimated Study Parameters (total of 571 screens)

MRI			MMG	
	S_e_, exact 95% CI	S_p_, exact 95% CI	S_e_, exact 95% CI	S_p_, exact 95% CI
All screening	84.6, 54.6−98.1	95.3, 93.3−96.9	23.1, 5.0−53.8	96.4, 94.5−97.8

## Discussion

Review of our institutional experience since the mid-2000’s in screening breast cancer survivors using MRI as an adjunct to mammography reveals that this imaging modality appears to have been useful in detecting early in-breast recurrences and/or contralateral primaries that might otherwise not have been detected at this early stage. In total, 7 of 10, or 70%, of screen-detected early cancers were mammographically-occult. It is interesting to consider these findings in light of the lack of official guidelines in favor or against MRI in this; following the recommendation to use mammography alone may have potentially missed these early cancers.

There is still a paucity of published data to support the use of MRI in this population; however there has been emerging retrospective, single-institution data that supports what we found at the University of Maryland, i.e. that MRI tends to detect early-stage tumors that may not have been discovered using mammogram alone. These studies are reviewed below (Table 5) (Liu et al. 2013; Gweon et al. 2013; Geiss et al. 2013; Brennan et al. 2010). Reported sensitivity of MRI is 91.8 and 83.3%; reported specificity is 92.2 and 98% in these studies (Liu et al. 2013; Geiss et al. 2013), with positive predictive values (PPVs) ranging from as low as 27% to as high as 59.2% (Liu et al. 2013; Gweon et al. 2013; Geiss et al. 2013; Brennan et al. 2010).

**Table 5:** Cancer detection using MRI in breast cancer survivors, review of the literature

Authors	No. of pts	Cancers/DCIS detected on MRI	Sensitivity	Specificity	PPV
Liu et al. (2013)	798	45	91.8%	92.2%	59.2%
Gweon et al. (2013)	704	10	83.3%	98%	41.7%
Geiss et al. (2013)	1,498	27^a^	n/a	n/a	27%
Brennan et al. (2010)	144	18	n/a	n/a	39%
Schacht et al. (2014)	208	6	n/a	n/a	n/a

^a^The study included mutation carriers; 7 of the 27 patients (22%) were known BRCA1 or BRCA2 mutation carriers.

Although there is a suggestion of benefit based on these experiences, and based on review of our own data, there is still much research to be done in the prospective setting before this modality can be widely recommended. One of the major limitations of aggressive surveillance in this population that has not rigorously been assessed is how the early detection of breast cancers impacts survival. Women who have survived early stage breast cancer do have competing mortality from the original tumor for several years after the diagnosis. In our study the MRI-detected breast tumors recurred at a median of 8.6 years following diagnosis of the original cancers (range 2–20). Thus it is not known if the detection of second primaries or of in-breast recurrences at early stages confers the same long-term survival benefit as early detection of first cancers in unaffected women. There is limited data on the impact of early detection of new tumors in this cohort, although there is some evidence that it may have some impact on survival (Houssami et al. 2009). Conversely, the overall 5-year mortality from breast cancer has continued to improve over recent years, making recurrences likely to become increasingly relevant as women live longer after their original diagnoses.

When comparing the use of breast MRI in this population, it is also useful to look at the cancer detection rate, which in our case was 4.4%. This rate is comparable to cancer detection rates in the high risk BRCA1 and BRCA2 mutation carrier population, for whom screening breast MRI has indeed been found to be useful and is an imaging modality recommended for wide usage. Although cross-study comparisons are not directly applicable due to differences in methodologies, the breast cancer detection rates in prospective cohorts of BRCA mutation carriers was actually slightly lower than in ours, ranging from 0.8 to 1.6% (Kriege et al. 2006; Leach et al. 2005; Kuhl et al. 2005; Lehman et al. 2005; Sardanelli et al. 2011; Kuhl et al. 2010). Additionally, in a retrospective study that directly compared the use of MRI in patients with a strong family history vs. patients with a personal history of breast cancer, the detection rate for the latter was higher (Schacht et al. 2014).

Our study has several limitations. The retrospective nature of the review of our institutional experience means that we did not control for bias in referral patterns and in selection of breast cancer survivors included in MRI surveillance; there may be inherent differences in our patients that may modify their risk profiles accordingly. Additionally, while we did identify two instances of interval cancers that were known to have occurred in this cohort, there was a small proportion of patients who were followed at outside centers and only referred to our tertiary center for breast imaging. On these patients, definitive follow-up data was not readily available and other interval cancers may have developed in these women that were not accounted for in our study. We also included studies done on patients as early as 2005. There is evidence that sensitivity of MRI improves over time with continued use at the same center, for example from 74 to 94% (*P* = 0.05) in one published experience (Passaperuma et al. 2012); thus early institutional experience may be less applicable than current results. Additionally, film mammography was discontinued in favor of digital studies in early 2008, and this may also have had an unknown effect on ongoing sensitivity.

We do note that almost 50% of patients included in our study were African-American. This is a patient population that may be excluded from other centers’ analyses due to referral patterns. African-American women are known to have lower incidence but higher mortality from breast cancer than Caucasians, even when accounting for possible differences in access to care and in screening practices (Curtis et al. 2008; Carey et al. 2006; Stark et al. 2010). The inclusion of this diverse patient population helps make our results translatable into real-world practices, whereas patient heterogeneity may make other studies’ results less applicable.

## Conclusion

Breast MRI as an adjunct to mammography improves detection of malignancy in breast cancer survivors. The cancer detection rate of 4.4% in our study was similar to the rate reported in BRCA mutation carriers, which is an established indication for using screening breast MRI as adjunct to mammography. While awaiting results of prospective studies in cohorts of breast cancer survivors, data from studies like ours may help guide management of patients in this population.
